# Corrigendum to TREM-2 promotes Th1 responses by interacting with the CD3ζ-ZAP70 complex following *Mycobacterium tuberculosis* infection

**DOI:** 10.1172/JCI213714

**Published:** 2026-11-02

**Authors:** Yongjian Wu, Minhao Wu, Siqi Ming, Xiaoxia Zhan, Shengfeng Hu, Xingyu Li, Huan Yin, Can Cao, Jiao Liu, Jinai Li, Zhilong Wu, Jie Zhou, Lei Liu, Sitang Gong, Duanman He, Xi Huang

Original citation: *J Clin Invest*. 2021;131(17):e137407. https://doi.org/10.1172/JCI137407

Citation for this corrigendum: *J Clin Invest*. 2026;136(21):e213714. https://doi.org/10.1172/JCI213714

The authors recently became aware that in Supplemental Figure 14G, the H&E image for the TREM-2^+^ group was incorrect. The supplemental PDF has been updated online with the correct figure.

The authors regret the error.

## Version 1 11/02/2026

Electronic publication

